# Acute Adverse Effects of Vaccines Against SARS-COV-2

**DOI:** 10.7759/cureus.27379

**Published:** 2022-07-27

**Authors:** Amina Mahmood, Syeda Ayat Shujaat, Meryum Hayat, Farhat Ijaz, Shanzay Habib, Waliya Sadaqat, Rana Khurram Aftab, Syed Hashim Ali Inam

**Affiliations:** 1 Medicine, CMH (Combined Military Hospital) Lahore Medical College and Institute of Dentistry, Lahore, PAK; 2 Physiology, CMH (Combined Military Hospital) Lahore Medical College and Institute of Dentistry, Lahore, PAK; 3 Medicine, Punjab Institute of Cardiology, Lahore, PAK; 4 Neurology, Marshall University Joan C. Edwards School of Medicine, Huntington, USA

**Keywords:** adverse side effect, sinopharm, vaccines, covid-19, sars-cov-2

## Abstract

Introduction

The global struggle against the impact of severe acute respiratory syndrome coronavirus 2 (SARS-COV-2) on physical and mental health and on economic and social aspects of human life continues even after two and a half years have passed since the emergence of this virus. The development of vaccines was a milestone. By June 2022, billions of people have been vaccinated against the deadly virus. However, like any other vaccine, the various vaccines against coronavirus disease 2019 (COVID-19) also cause a variety of adverse effects. Therefore this study aimed to determine the different acute side effects experienced after receiving the vaccines and correlating them with some socio-demographic and biomedical factors.

Methodology

This cross-sectional study has a sample size of 467. Study participants were recruited after fulfilling inclusion and exclusion criteria. After gaining approval from the Ethical Review Board (ERB) of CMH Lahore Medical College and Institute of Dentistry, Lahore, Pakistan, an online questionnaire was distributed via social media. The survey questionnaire had a series of questions regarding the socio-demographic and biomedical characteristics of the participants, as well as the type of vaccine they got, followed by questions about the development of adverse effects after each dose (first and second). Data were statistically analyzed using IBM SPSS Statistics for Windows, Version 25.0 (Released 2017; IBM Corp., Armonk, New York, United States). The analysis was carried out in a confidence range of 95%, and a p-value<0.05 was considered statistically significant.

Results

Sinopharm (76.0%) was the most frequently received vaccine. Adverse events were reported more after the first dose (79.7%) than in the second (67.2%) (p value 0.001). The reported adverse events after either dose were of mild intensity (p<0.05). None of the individuals reported serious adverse events or hospitalization after getting the shots. Females, younger age groups, and individuals with BMI in the underweight category were more prone to developing symptoms and experiencing difficulty doing routine work after getting the doses. The associations were statistically significant (p<0.05). Blood group (A,B,0,AB), past COVID-19 history, and smoking status were not positively associated with the appearance of symptoms after either dose or with inconvenience doing daily work post-vaccination.

Conclusion

The vaccines developed against COVID-19 offer benefits that outweigh the few mild adverse effects experienced. None of these symptoms is severe enough to stop an individual from doing routine work or result in morbidity or mortality. Therefore, people should avoid any hesitancy towards getting vaccinated to get past this pandemic.

## Introduction

WHO declared the Coronavirus disease 2019 (COVID-19) outbreak a pandemic in March 2020 [1]. More than two years later, the world is still facing the devastating effects of this pandemic. As of July 1, 2022, there have been 545,226,550 confirmed coronavirus cases, including 6,334,728 deaths worldwide [2]. Just like the world, Pakistan is facing challenges during this period, like the struggle to contain the rising cases of COVID-19 and enforce lockdowns and restrictions. These unprecedented circumstances have not only had a toll on people's mental health but have also led to increasing unemployment, learning losses, and domestic violence [3].

There was needed to develop a preventive COVID-19 vaccine without compromising safety and efficacy. On April 9, 2020, WHO released target product profiles for COVID-19 vaccines guiding clinical trial design, implementation, evaluation, and follow-up. Several preclinical and clinical trials started for different vaccines [4]. According to WHO, by February 18, 2021, at least seven other vaccines against coronavirus had been rolled out worldwide, with 200 additional vaccine candidates in different phases of development. 

Pakistan started its vaccination drive in February 2021, prioritizing frontline healthcare workers and senior citizens [5]. More than 41.5 million doses of the COVID-19 vaccine have been administered in Pakistan, with 5.4% of the population fully vaccinated and 9.8% partly vaccinated [6]. The vaccines administered across Pakistan are Sinopharm, Sinovac, Pakvac, Cansino, Astrazeneca, Sputnik V, Pfizer-BioNTech, and Moderna.

Pakistan has ramped up its vaccination drive, especially in Punjab, and launched a door-to-door coronavirus vaccination campaign. However, it has been reported globally and locally in Pakistan that there is COVID-19 vaccine hesitancy among people due to multiple reasons, ranging from religious factors, myths, conspiracy theories, and especially fears of adverse effects [7].

Previously, several clinical trials and a few surveys on the adverse effects of different COVID-19 vaccines on populations other than Pakistan's have been published [8]. To the best of our knowledge, no studies have been done to know the acute adverse effects experienced by the general Pakistani population getting vaccinated against COVID-19. No previous study assessed how different demographic and biomedical factors are associated with these negative effects. Furthermore, reassurance to the Pakistani population about the safety of the COVID-19 vaccine by evaluating its adverse effects is the need of the hour to improve vaccine acceptance because negative perceptions and fears among the population toward COVID-19 vaccines would have devastating implications regarding efforts to end the pandemic.

## Materials and methods

This cross-sectional questionnaire-based survey has a sample size of 467 and was conducted from December 2021 to March 2022. A convenient non probability sampling technique was used. All individuals aged 20 years or above, from either gender, who had received both doses of the COVID-19 vaccine were included. Pregnant patients, those with diabetic and hypertensive profiles, known asthmatics, immunocompromised patients, individuals unwilling for vaccination, those who had a COVID-19 disease history in the last three months, individuals allergic to drug contents, or individuals having a specific contraindication to vaccinations were excluded from this study. After gaining the approval of the Ethical Review Board (ERB) of CMH Lahore Medical College and Institute of Dentistry, Lahore, Pakistan (54/ERC/CMHLMC), this survey was conducted via online Google Forms (Google LLC, Mountain View, California, United States). Participants were provided with adverse effects online proforma devised by relevant studies on this subject. The questionnaire consisted of three sections. The first section included consent and questions regarding socio-demographic data (age, gender), blood group, weight, height, history of COVID-19, smoking status, and the type of vaccine administered. The second section asked a series of questions regarding the local and systemic adverse effects experienced after receiving dose one and their impact on daily activities. The third section had the same line of questions for the second dose. Any of the participants experiencing any of these complaints after first and second dose, e.g., injection site pain, muscle pain, fatigue, headache, injection site redness, injection site swelling, chills, joint pain, fever, nausea, vomiting, diarrhea, sore throat, and swollen lymph nodes were labelled as post dose symptoms. Inconvenience was defined as how the symptoms might have affected their ability to do daily work and carry out their routine tasks and if the symptoms were strong enough to stop them from carrying out their tasks

Statistical analysis

Data were statistically analyzed using IBM SPSS Statistics for Windows, Version 25.0 (Released 2017; IBM Corp., Armonk, New York, United States). The relationship of all variables was studied with the appearance of adverse effects after doses one and two and with inconvenience during routine work after receiving the doses. Qualitative variables were presented as frequency and percentage; Chi-square test was applied for comparison between variables. The study was conducted in a confidence range of 95%, and a p-value<0.05 was considered statistically significant. 

## Results

Of the 467 participants, 156 (33.4%) were males, and 311 (66.6%) were females. 355 (76.0%) individuals had received Sinopharm, followed by Sinovac, which was received by 86 (18.4%) subjects. Inoculation of Sputnik (3.2%), Moderna (1.5%), AstraZeneca (0.6%) and Pfizer (0.2%) were less frequently reported. 

Participants reported symptoms more frequently after dose 1 (372, 79.7%) than dose 2 (314, 67.2%). Inconvenience doing routine work was experienced by 13.1% of participants after dose 1 and 9.9% after dose 2. The most common side effects developed post the first shot was injection site pain (66.6%), muscle pain (49.9%), fatigue (48.4%), and headache (33.4%). Injection site pain (54.6%), fatigue (34.9%), muscle pain (31.3%), and headache (23.8%) were also the most often reported symptoms after the second shot. Most people had a mild form of these side effects (p=0.001). Injection site redness, swelling, chills, joint pain, fever, nausea, vomiting, diarrhoea, sore throat, and swollen lymph nodes were rarely reported after either dose. None of the subjects reported a severe reaction or hospitalization after receiving the doses. 

A statistically significant association was seen between age group and development of above mentioned symptoms after dose 1 with the highest frequency (92.3%) in the 31-40 years age group (p=0.001) (Table 1). The age group of 18-30 years was more likely to face inconvenience while doing routine work after dose 1 (p=0.001) (Table 1). A positive association was observed between gender and the appearance of symptoms after dose 1 (Table 1) and dose 2 (Table 2), with females being more prone (p=0.001). Females also reported inconvenience in doing daily work more often than their male counterparts after both the doses and the difference was statistically significant (p=0.001) (Tables 1, 2). Inconvenience doing routine work after dose 1 (p=0.007) (Table 1), development of symptoms after dose 2 (p=0.001), and inconvenience after the second dose (p=0.001) (Table 2) were more often reported by individuals with a BMI in the underweight range as compared to others. 

**Table 1:** Symptoms and inconvenience post dose 1 by age, gender, BMI, smoking status, past COVID-19 history, and blood groups COVID-19: coronavirus disease 2019

DOSE 1								
Symptoms post dose 1					Inconvenience post dose 1			
		Yes	No	P-value	Yes	No	No symptoms	P-value
Age	18-30 (%)	294 (84.0)	56 (16.0)	0.001*	57 (16.3)	238 (68.0)	55(15.7)	0.001*
	31-40 (%)	24 (92.3)	2 (7.7)		3* (11.5)	21 (80.8)	2 (7.7)	
	41-50 (%)	20 (66.7)	10 (33.3)		0* (0)	20 (66.7)	10 (33.3)	
	51-60 (%)	19 (65.5)	10 (34.5)		1* (3.4)	18 (62.1)	10 (34.5)	
	61-70 (%)	13 (48.1)	14 (51.9)		0* (0)	13 (48.1)	14 (51.9)	
	71-80 (%)	2 (40.0)	3 (60.0)		0* (0)	2* (40.0)	3* (60.0)	
Gender	Male (%)	109 (69.9)	47 (30.1)	0.001*	13 (8.3)	97 (62.2)	46 (29.5)	0.001*
	Female (%)	263 (84.6)	48 (15.4)		48 (15.4)	215 (69.1)	48 (15.4)	
BMI	Underweight (%)	42 (80.8)	10 (19.2)	0.31	9 (17.3)	34 (65.4)	9 (17.3)	0.007*
	Normal (%)	212 (81.9)	47 (18.1)		39 (15.1)	173 (66.8)	47 (18.1)	
	Overweight (%)	85 (78.0)	24 (22.0)		11 (10.1)	74 (67.9)	24 (22.0)	
	Obese (%)	33 (70.2)	14 (29.8)		2 (4.3)	31 (66.0)	14 (29.8)	
Smoking Status	Smoker (%)	19 (65.5)	10 (34.5)	0.051	5 (17.2)	14 (48.3)	10 (34.5)	0.075
	Non-smoker (%)	353 (80.6)	85 (19.4)		56 (12.8)	298 (68.0)	84 (19.2)	
Past COVID-19 history	Yes (%)	79 (82.3)	17 (17.7)	0.472	14 (14.6)	65 (67.7)	17 (17.7)	0.748
	No (%)	293 (79.0)	78 (21.0)		47 (12.7)	247 (66.6)	77 (20.8)	
Blood group	A (%)	77 (72.0)	30 (28.0)	0.078	14 (10.2)	63 (46.0)	60 (43.8)	0.174
	B (%)	136 (78.2)	38 (21.8)		20 (11.5)	117 (67.2)	37 (21.3)	
	O (%)	97 (86.6)	15 (13.4)		19 (17.0)	78 (69.6)	15 (13.4)	
	AB (%)	43 (82.7)	9 (17.3)		4 (7.7)	39 (75.0)	9 (17.3)	
	Unknown (%)	19 (86.4)	3 (13.6)		4 (18.2)	15 (68.2)	3 (13.6)	

**Table 2:** Symptoms and inconvenience post dose 2 by age, gender, BMI, smoking status, past COVID-19 history, and blood groups COVID-19: coronavirus disease 2019

DOSE 2								
	Symptoms post dose 2				Inconvenience post dose 2			
		Yes	No	P-value	Yes	No	No symptoms	P-value
Age	18-30	245 (70.0)	105 (30.0)	0.102	42 (12.0)	205 (58.6)	103 (29.4)	0.075
	31-40	19 (73.1)	7 (26.9)		1*(3.8)	18 (69.2)	7 (26.9)	
	41-50	16 (53.3)	14 (46.7)		1*(3.3)	15 (50.0)	14 (46.7)	
	51-60	18 (62.1)	11 (37.9)		1* (3.4)	16 (55.2)	12 (41.4)	
	61-70	13 (48.1)	14 (51.9)		0* (0)	13 (48.1)	14 (51.9)	
	71-80	3 (60.0)	2 (40.0)		1* (20.0)	2* (40.0)	2* (40.0)	
Gender	Male	83 (53.2)	73 (46.8)	0.001*	6 (3.8)	77 (49.4)	73 (46.8)	0.001*
	Female	231 (74.3)	80 (25.7)		40 (12.9)	192 (61.7)	79 (25.4)	
BMI	Underweight	39 (75.0)	13 (25.0)	0.001*	9 (17.3)	30 (57.7)	13 (25.0)	0.001*
	Normal	183 (70.7)	76 (29.3)		33 (12.7)	150 (57.9)	76 (29.3)	
	Overweight	70 (64.2)	39 (35.8)		2 (1.8)	68 (62.4)	39 (35.8)	
	Obese	22 (46.8)	25 (53.2)		2 (4.3)	21 (44.7)	24 (51.1)	
Smoking status	Smoker	5 (26.3)	14 (73.7)	0.066	2 (6.9)	13 (44.8)	14 (48.3)	0.174
	Non-smoker	299 (68.3)	139 (31.7)		44 (10.0)	256 (58.4)	138 (31.5)	
Past COVID-19 history	Yes	63 (65.6)	33 (34.4)	0.706	9 (9.4)	54 (56.3)	33 (34.4)	0.909
	No	251 (67.7)	120 (32.3)		37 (10.0)	215 (58.0)	119 (32.1)	
Blood group	A (%)	72 (67.3)	35 (32.7)	0.158	11 (10.3)	61 (57.0)	35 (32.7)	0.495
	B (%)	122 (70.1)	52 (29.9)		16 (9.2)	106 (60.9)	52 (30.0)	
	O (%)	77 (68.8)	35 (31.3)		12 (10.7)	65 (58.0)	35 (31.3)	
	AB (%)	27 (51.9)	25 (48.0)		3 (5.8)	25 (48.1)	24 (46.2)	
	Unknown (%)	16 (72.7)	6 (27.3)		4 (18.2)	12 (54.5)	6 (27.3)	

## Discussion

With the COVID-19 vaccine in Pakistan, many people have hesitancy to get vaccinated due to fear of side effects. Although local and systemic reactions are expected and often transient, they may influence the patients' perception of vaccine administration. Thus, this study aims to analyze the adverse effects experienced by the general population of Pakistan after getting vaccinated against COVID-19 and tries to assess the different demographic factors associated with these adverse effects. 

Various COVID-19 vaccines have been administered among the participants. It has been proven that the inactivated SARS-CoV-2 vaccines have reported fewer adverse effects compared to other candidate vaccines [9]. The negative impact of the Sinopharm BBIBP-CorV was 29% in the phase 1 clinical trial and 23% in the phase 2 clinical trial. After the assessment of Sinopharm by WHO, moderate confidence was established in the finding that the risk of serious adverse events following one or two doses of Sinopharm in adults (18-59 years) is low. While most adverse effects range from mild to moderate, the common ones include pain at the injection site, fatigue, and headache [10].

In our study, 79.7% of participants experienced side effects following the first dose, while the remaining 20.3% reported no adverse effects at all. Similarly, after administering the second dose, most participants (67.2%) reportedly experienced side effects, with the remaining 32.8% experiencing none. According to the Centers for Disease Control and Prevention (CDC), the common adverse effects of COVID-19 vaccines include pain, swelling, and redness at the injection site, as well as fatigue, chills, fever, myalgia, headache, and nausea. Even though more people reported side effects after the first dose, the CDC states that the side effects experienced after the second dose may be more intense [11].

Women reported more side effects than men, noticeable with vaccines like Pfizer and Moderna, though people receiving AstraZeneca and Pfizer in our study were too small. Same findings were reported in another study [12]. Although there isn't sufficient data comparing gender-based immune responses to the COVID-19 vaccine, researchers from a previous study found that women underwent more significant cytokine and antibody responses compared with men after getting the flu vaccine [13]. This development of a more significant immune response to vaccines by women and differences in hormones between the two genders might influence the immune responses. However, more research needs to be conducted to determine whether testosterone plays a significant role in this difference or not.

Inconvenience doing routine work after dose 1 (p=0.007) and inconvenience after dose 2 (p=0.001) is more often reported by individuals with a BMI in the underweight range than others. Low BMI is associated with a lack of proper nutrition, with deficiencies in calcium and iron leading to osteoporosis and anemia. Furthermore, not getting sufficient calories to obtain a healthy weight can lead to significant elevation in the levels of fatigue and tiredness in these individuals, thus accounting for the inconvenience in carrying out simple routine work.

With increasing age, our immune system weakens, and the reactogenicity decreases. However, younger people below 50 who have a healthier immune system can develop a rapid and robust immune response to vaccines, which may inevitably lead to more adverse effects [14]. This study found that adverse effects experienced by the 18-30 and 31-40 age groups after both doses are greater than those experienced by the higher age groups. 

## Conclusions

Injection site pain, muscle pain, fatigue, and headache were the common site effects. To have a clearer picture of the side effects of Covid-19 vaccines, multicenter studies with large sample sizes are required. The vaccines developed against COVID-19 offer benefits that outweigh the few mild adverse effects experienced. None of these symptoms is severe enough to stop an individual from doing routine work or result in morbidity or mortality. Therefore, people should avoid any hesitancy towards getting vaccinated to get past this pandemic.
